# Dose-dense epirubicin and paclitaxel with G-CSF: a study of decreasing intervals in metastatic breast cancer

**DOI:** 10.1054/bjoc.2000.1202

**Published:** 2000-05-18

**Authors:** R I Lalisang, E E Voest, J A Wils, J W Nortier, F L Erdkamp, H F Hillen, J Wals, H C Schouten, G H Blijham

**Affiliations:** Departments of Internal Medicine, Maastricht University Hospital, PO Box 5800, Maastricht, AZ, 6202; Utrecht University Hospital, PO Box 85500, Utrecht, GA, 3508; St. Laurentius Hospital, PO Box 620, Roermond, AX, 6040; Diaconessen Hospital, PO Box 80250, Utrecht, GA, 3508; Maasland Hospital, PO Box 5500, Sittard, MB, 6130; Atrium Medical Centre, PO Box 255, Brunssum, AG, 6440, The Netherlands

**Keywords:** combination chemotherapy, breast neoplasms, dose-intensive, epirubicin, paclitaxel

## Abstract

Anthracyclines and taxanes are very effective drugs in the treatment of advanced breast cancer. With G-CSF support, the dose-intensity of this combination can be increased by reducing the interval between chemotherapy cycles, the so-called ‘shortening of cycle time’. We treated 36 patients with advanced breast cancer in a multicentre phase I/II study. The treatment regimen consisted of epirubicin 75 mg m^−2^followed by paclitaxel 135 mg m^−2^(3 h) in combination with G-CSF. At least six patients were treated in each cohort and were evaluated over the first three cycles. Starting at an interval of 14 days, in subsequent cohorts of patients the interval could be shortened to 10 days. An 8-day interval was not feasible due mainly to incomplete neutrophil recovery at the day of the next scheduled cycle. In the 10-day interval cohort it was feasible to increase the paclitaxel dose to 175 mg m^−2^. The haematological and non-haematological toxicity was relatively mild. No cumulative myelosuppression was observed over at least three consecutive cycles. In combination with G-CSF, epirubicin 75 mg m^−2^and paclitaxel 175 mg m^−2^could be safely administered every 10 days over at least three cycles, enabling a dose intensity of 52 and 122 mg m^−2^per week, respectively. © 2000 Cancer Research Campaign


*British Journal of Cancer* (2000) **82**(12), 1914–1919
© 2000 Cancer Research Campaign
doi: 10.1054/ bjoc.2000.1202, available online at http://www.idealibrary.com on IDEAL®

### Dose-dense epirubicin and paclitaxel with G-CSF: a study of decreasing intervals in metastatic breast cancer

**RI Lalisang[1], EE Voest[2], JA Wils[3], JW Nortier[4], FL Erdkamp[5], HF Hillen[1], J Wals[6], HC Schouten[1] and GH Blijham[2]**

From the Departments of Internal Medicine, [1]Maastricht University Hospital, PO Box 5800, 6202 AZ, Maastricht; [2]Utrecht University Hospital, PO Box 85500, 3508 GA, Utrecht; [3]St. Laurentius Hospital, PO Box 620, 6040 AX, Roermond; [4]Diaconessen Hospital, PO Box 80250, 3508 GA, Utrecht; [5]Maasland Hospital, PO Box 5500, 6130 MB, Sittard; [6]Atrium Medical Centre, PO Box 255, 6440 AG, Brunssum, The Netherlands

**Summary** Anthracyclines and taxanes are very effective drugs in the treatment of advanced breast cancer. With G-CSF support, the dose-intensity of this combination can be increased by reducing the interval between chemotherapy cycles, the so-called 'shortening of cycle time'. We treated 36 patients with advanced breast cancer in a multicentre phase I/II study. The treatment regimen consisted of epirubicin 75 mg m$^{-2}$ followed by paclitaxel 135 mg m$^{-2}$ (3 h) in combination with G-CSF. At least six patients were treated in each cohort and were evaluated over the first three cycles. Starting at an interval of 14 days, in subsequent cohorts of patients the interval could be shortened to 10 days. An 8-day interval was not feasible due mainly to incomplete neutrophil recovery at the day of the next scheduled cycle. In the 10-day interval cohort it was feasible to increase the paclitaxel dose to 175 mg m$^{-2}$. The haematological and non-haematological toxicity was relatively mild. No cumulative myelosuppression was observed over at least three consecutive cycles. In combination with G-CSF, epirubicin 75 mg m$^{-2}$ and paclitaxel 175 mg m$^{-2}$ could be safely administered every 10 days over at least three cycles, enabling a dose intensity of 52 and 122 mg m$^{-2}$ per week, respectively. © 2000 Cancer Research Campaign

**Keywords:** combination chemotherapy; breast neoplasms; dose-intensive; epirubicin; paclitaxel

Anthracyclines and taxanes are amongst the most active single agents in advanced breast cancer. Studies of single agent paclitaxel demonstrate that leucopenia is frequent and dose-limiting in all schedules (3-, 6-, 24-h infusion) evaluated. Randomized trials evaluating the 3- and/or 24-h schedules, each with two paclitaxel doses of 135 and 175 mg m$^{-2}$, have revealed that neutropenia is dose- and schedule-dependent, without apparent difference in efficacy (Eisenhauer et al, 1994; Nabholtz et al, 1996; Gianni et al, 1995*a*). The optimal therapeutic schedule of paclitaxel is still unknown and, on practical grounds, the short infusion over 3 h is safe, convenient and effective (Gianni, 1995*b*).

Paclitaxel at longer infusion rate (≥ 24 h) has been given in combination with doxorubicin. The maximal tolerable dose (MTD) and toxicity profile (primarily mucositis and neutropenia) of this combination appear to depend on the sequence and infusion duration of the two drugs (Fisherman et al, 1993; Sledge Jr et al, 1994). In a study of escalating doses paclitaxel (3-h infusion) in combination with fixed dose of doxorubicin (60 mg m$^{-2}$ i.v. bolus) in a 3-weekly schedule, mucositis, long-lasting grade 4 neutropenia and febrile neutropenia defined the MTD of paclitaxel at 200 mg m$^{-2}$, and was not sequence dependent. This regimen was very effective in chemotherapy-naive breast cancer patients, but at the cost of considerable increased cardiotoxicity (Gianni et al, 1995*c*; Gehl et al, 1996). 4′-Epi-doxorubicin (epirubicin) is a synthetic doxorubicin analogue, with similar anti-tumour activity as doxorubicin at equimolar doses, but decreased overall toxicity, in particular cardiotoxicity (Brambilla et al, 1986; Jain et al, 1985). It may, therefore, be an attractive substitute for doxorubicin in combination with taxanes.

*Received 7 April 1999*
*Revised 23 November 1999*
*Accepted 24 February 2000*

*Correspondence to:* RI Lalisang

Granulocyte colony stimulating factor (G-CSF) can be used to ameliorate neutropenia and to obtain increased dose-intensity by allowing a higher dose of chemotherapy per cycle (dose-escalation) or by allowing a shortening of the interval between cycles (dose-dense). Both approaches may lead to a higher dose-intensity, but their biological effect and clinical relevance may be quite different (Henderson et al, 1988). Dose-dense chemotherapy may be important to overcome the cellular cytokinetic resistance of tumors (Gilewski and Norton, 1996). Dose-escalation is based on a steep dose–response relationship, whereby the doses of chemotherapy are increased up to the limits of haematological and non-haematological toxicity. These two approaches were investigated by our group in the treatment of metastatic breast cancer with epirubicin and cyclophosphamide. With the addition of G-CSF, interval reduction permitted a higher dose-intensity, with less toxicity, than dose escalation (Lalisang et al, 1997). Based on these results we started a study with the aim of increasing the dose-intensity of a conventional dose of epirubicin (75 mg m$^{-2}$ i.v. bolus) and paclitaxel (135 mg m$^{-2}$ 3-h infusion) combination by shortening of the cycle time. We intended to define the minimal tolerable interval of this chemotherapy regimen in combination with G-CSF support. During the study the protocol was amended, and allowed testing a higher paclitaxel dose of 175 mg m$^{-2}$ in the shortest feasible interval. The second aim was to assess the safety profile of this approach.

### PATIENTS AND METHODS

### Patient selection

The study protocol was approved by the institutional review boards of the participating hospitals, and patients gave informed consent. Women with advanced breast cancer had to fulfil the following inclusion criteria:

- Histological proof of breast cancer
- Age 18–70 years
- Performance status 0–2 (Eastern Cooperative Oncology Group scale)
- Neutrophil count ≥ 2 × $10^9$ $l^{-1}$ and platelet count ≥ 100 × $10^9$ $l^{-1}$
- Adequate function tests for liver (bilirubin level < 25 µmol $l^{-1}$ and transaminase level (< 3 × upper limit of normal) and kidneys (serum creatinine level < 150 µmol $l^{-1}$)
- No prior chemotherapy for metastatic disease
- Prior adjuvant chemotherapy allowed, if interval last chemotherapy cycle ≥ 1 year and at entry lifetime cumulative dose of doxorubicin ≤ 300 mg $m^{-2}$ and epirubicin ≤ 450 mg $m^{-2}$
- Prior radiotherapy involving ≤ 25% of red bone marrow
- Left ventricle ejection fraction (LVEF) by multigated (MUGA) isotope cardiograph ≥ 50% and without symptomatic cardiovascular disease
- No central nervous system involvement.

### Treatment plan

In this schedule-finding study, the epirubicin (Pharmacia & Upjohn, Milan, Italy), paclitaxel (Taxol, Bristol-Myers Squibb Pharmaceuticals, Princeton, NJ, USA) as well as R-metHuG-CSF (Filgrastim, Amgen Inc., Thousands Oaks, CA, USA) doses were kept constant, and four intercyclic intervals were foreseen. The starting interval of 14 days was planned to be decreased to 12, 10 and 8 days, respectively. To prevent hypersensitivity reactions due to paclitaxel, a routine premedication regimen was adopted: oral or intravenous dexamethasone 20 mg (6 and 12 h pre-treatment); clemastine 2 mg and ranitidine 50 mg both intravenously 30–60 min before paclitaxel administration. Epirubicin was given as a short i.v. infusion on day 1 at a fixed dose of 75 mg $m^{-2}$. Paclitaxel at a dose of 135 mg $m^{-2}$ was administered by a 3-h infusion, starting 5 min after epirubicin administration. G-CSF (300 µg for patients ≤ 70 kg and 480 µg for patients > 70 kg) was administered once daily subcutaneously on all days except the days of chemotherapy.

A cohort of at least six patients was studied at each interval. The neutrophil (≥ 2 × $10^9$ $l^{-1}$) and platelet (≥ 100 × $10^9$ $l^{-1}$) counts had to have recovered on the day of scheduled chemotherapy. For this study specific dose-intensity limiting criteria (DILC, see Table 1) had been defined. Proceeding to the next, shorter, interval level was only done after completion of the previous cohort and if less than 50% of those patients had experienced a DILC during the first three courses. In case of incomplete haematological recovery treatment was delayed. Concomitant hormonal therapy or prophylactic antibiotic therapy was not allowed. Patients were transfused when necessary to maintain a platelet count of ≥ 15 × $10^9$ $l^{-1}$ and haemoglobin level ≥ 6 mmol $l^{-1}$.

The patients had to complete a minimum of three cycles, except if one of the following events occurred: disease progression, DILC, any other unacceptable toxicity precluding further therapy, or patients' refusal to continue treatment. After completion of the first three cycles further therapy was left to the discretion of the investigator.

Table 1 Dose-intensity limiting criteria

| | |
|---|---|
| 1 | Any WHO grade 3 or 4 non-haematological toxicity |
| 2 | Neutropenia grade 4: neutrophil count < 0.5 × $10^9$ $l^{-1}$ for a period of more than 7 days |
| 3 | Febrile neutropenia: neutrophil count < 0.5 × $10^9$ $l^{-1}$ and fever |
| 4 | Thrombocytopenia grade 4: platelet count < 25 × $10^9$ $l^{-1}$ for more than 4 days |
| 5 | Delay of chemotherapy due to incomplete recovery on the day of scheduled therapy:<br>Haematological: neutrophil count < 2 × $10^9$ $l^{-1}$ and/or platelet count < 100 × $10^9$ $l^{-1}$<br>Persistence of non-haematological side-effects of WHO grade 2 or more (excluding alopecia and anticipatory nausea and vomiting) |
| 6 | Cardiotoxicity, defined by development of clinical cardiac failure or an absolute decrease in MUGA LVEF ≥ 20% (EF absolute units) from baseline to a value above 50% or ≥ 10% (EF absolute units) to a value below 50% |

As the 8-day interval appeared to be not feasible (see Results) a new cohort was tested with a 10-day interval but using a higher paclitaxel dose of 175 mg $m^{-2}$.

The minimal tolerable interval (MTI) for this study was defined as the shortest interval that resulted in less than 50% instances of DILCs among treated patients in each cohort. To express the dose-intensity of the treatment the equation, delivered dose-intensity (DDI), the actually given dose per $m^2$ per week during the first three protocol cycles of treatment, was used.

### Pre-treatment and follow-up evaluation

All patients were initially evaluated with a history, physical examination, complete blood-cell count (CBC), liver and kidney function tests, ECG, LVEF MUGA scan, chest X-ray and bone scan. If indicated additional radiological examinations of suspected areas, with tumour measurements (if possible) were performed. CBC was repeated twice weekly and a biochemical profile was assessed before each cycle. Follow-up LVEF MUGA scan was requested after three cycles, in case of clinical signs of congestive heart failure, if patients went off study or at a cumulative dose of 500 mg $m^{-1}$, at 800 mg $m^{-2}$ of epirubicin and subsequently before each additional treatment course thereafter. In our study cardiac toxicity was defined as development of clinical cardiac failure and/or an absolute decrease in resting LVEF either ≥ 20% (EF absolute units) from baseline to a value above 50% or ≥ 10% to a value below 50%.

Tumour evaluation was repeated after each three cycles, at the end of treatment or as clinically indicated and evaluated according to UICC-criteria (Miller et al, 1981). Toxicity was assessed after each course according to WHO grading criteria (Miller et al, 1981) except for neutropenia, thrombocytopenia, febrile neutropenia and cardiotoxicity for which adjusted criteria were applied (Table 1).

### RESULTS

### Patient characteristics

Forty-one eligible patients were entered, and five patients were found to be not evaluable; four patients in the 8-day interval

© 2000 Cancer Research Campaign

**Table 2** Patient characteristics

| | |
|---|---|
| Eligible/evaluable for toxicity | 36 |
| Median age (range) | 51 (24–68) |
| Performance ECOG 0/1/2 | 14/18/4 |
| Adjuvant chemotherapy | 14 |
| Including anthracyclines | 5 |
| Previous chest/breast irradiation | 28 |
| Sites of disease in patients (% of all patients) | |
| Bone | 64% |
| Soft tissue | 81% |
| Lung | 36% |
| Liver | 47% |
| Lung and/or liver | 61% |
| No. of organs involved | |
| Median (range) | 2 (1–5) |

**Table 3** Epirubicin/Paclitaxel + G-CSF: Initial dose-intensity limiting criteria in the first three cycles

| Dose EP mg m$^{-2}$<br>Interval days | 75/135<br>14 | 75/135<br>12 | 75/135<br>10 | 75/135<br>8 | 75/175<br>10 |
|---|---|---|---|---|---|
| Evaluable patients | 6 | 8 | 10 | 6 | 6 |
| Patient(s) with DILC | 1 | 1 | 1 | 4 | 2 |
| DILC | | | | | |
| ANC-recovery | – | – | 1 | 3 | – |
| Platelet-recovery | 1 | – | – | – | |
| Febrile neutropenia | – | – | – | 1[a] | – |
| Mucositis ≥ grade 3 | – | – | – | 1[#] | – |
| Nausea/vomiting ≥ 3 | – | 1 | – | – | – |
| Infection | – | – | – | – | 1 |
| Cardiotoxicity | 1 | – | – | – | 1 |

[a] Same patient with concurrent febrile neutropenia and mucositis grade 3 after cycle 2

(protocol violations after cycle 1 [two patients] and central venous line complications [two patients]) and in the 10-day interval with paclitaxel 175 mg m$^{-2}$ one patient (too low G-CSF dose). Therefore, 36 patients were fully assessable for the present report. The characteristics of these 36 patients are shown in Table 2. These 36 patients received 131 cycles according to the protocol until DILC or protocol completion.

### Initial dose-intensity limiting criteria in the first three consecutive cycles

In this study analysing shortening of intervals with fixed doses of epirubicin and paclitaxel 75/135 mg m$^{-2}$, six patients were entered at the starting interval of 14 days. One patient had an incomplete platelet recovery at scheduled cycle 2 and a transient pathological decrease of the LVEF after cycle 2 (Table 3). At the 12-day interval one out of eight patients met a DILC: nausea/vomiting WHO grade 3 after the third cycle. In the 10-day interval one out of 10 patients had a DILC: an incomplete neutrophil recovery after cycle 3. At the 8-day interval four out of six patients met a DILC during the first three cycles, making this interval not feasible based on the predefined criteria. The initial DILCs were: incomplete neutrophil recovery at scheduled cycle 2 in three patients and combination of febrile neutropenia, pulmonary embolism and mucositis WHO grade 3 after cycle 2 in the fourth patient. Ten days was the shortest feasible interval for epirubicin/paclitaxel 75/135 mg m$^{-2}$ with G-CSF and the median DDI for the first three cycles was 52 (range 49–56) mg m$^{-2}$ per week and 94 (range 88–100) mg m$^{-2}$ per week, respectively.

To investigate whether the dose of paclitaxel could be increased in the 10-day interval epirubicin/paclitaxel 75/175 mg m$^{-2}$ was tested. Two out of six patients encountered a DILC, transient asymptomatic pathologic decrease in cardiac LVEF after the third course, and infection (pneumonia) grade 2 at scheduled cycle 4, respectively. The median DDI in the first three cycles was epirubicin 52 (52–53) mg m$^{-2}$ per week and paclitaxel 122 (117–126) mg m$^{-2}$ per week.

### Cumulative toxicity

In the 14-day interval all five patients without a DILC in the first three cycles continued at their scheduled interval for at least six cycles. In the 12-day interval seven out of eight patients continued the same chemotherapy combination after three protocol cycles, although only two patients at scheduled interval for a total of four cycles, all without additional toxicities.

In the 10-day interval three out of 10 patients continued scheduled treatment for a total of four (two patients) and nine (one patient) cycles, respectively. The single patient with an incomplete neutrophil recovery after cycle three developed a reversible neuropathy grade 3 after cycle 6. In the 8-day interval the two patients without a DILC in the initial three cycles continued the scheduled interval for a total of five and six cycles, respectively. The patient with a febrile neutropenia and stomatitis grade 3 after the second cycle also developed pulmonary embolism. In the 10-day interval at 75/175 mg m$^{-2}$ only one patient continued scheduled treatment for six cycles. The kinetics of neutrophil and platelet counts for the patients in the various intervals are shown in Figure 1. The non-haematological toxicitys were generally mild and are displayed in Table 4. Only one patient encountered a hypersensitivity reaction WHO grade 2.

### Transfusion of blood products

In this dose-dense schedule, during the first three cycles red blood-cell (RBC) transfusions were needed in five patients. Most transfusions occurred in the patients receiving more than three cycles (14 of 27 patients). Platelet transfusions were not given.

### Cardiotoxicity

A total of 98 MUGA scans were performed; basal and follow-up scans were available in 35 of 36 patients, making them evaluable for cardiotoxicity by ejection fraction. A pathological decline in LVEF, as defined earlier, was observed in two (6%) patients in the first three cycles, both not pre-treated with anthracyclines. The first patient (previously irradiated parasternally) was treated in the 14-day interval, developed a delayed platelet recovery after the first cycle, received cycle 2 after platelet recovery, which was complicated by severe dyspnoea and anaemia (4.5 mmol l$^{-1}$), with a transient decrease in LVEF (63→49%). On physical examination, chest X-ray and ECG no signs of congestive heart failure were observed. After RBC transfusion the patient recovered and a control LVEF showed normalization (62%). This patient continued sheduled treatment for an additional three cycles, without further signs of cardiac failure. A short interval (1 day) between irradiation of the lumbar spine and start of study treat-

 *© 2000 Cancer Research Campaign*

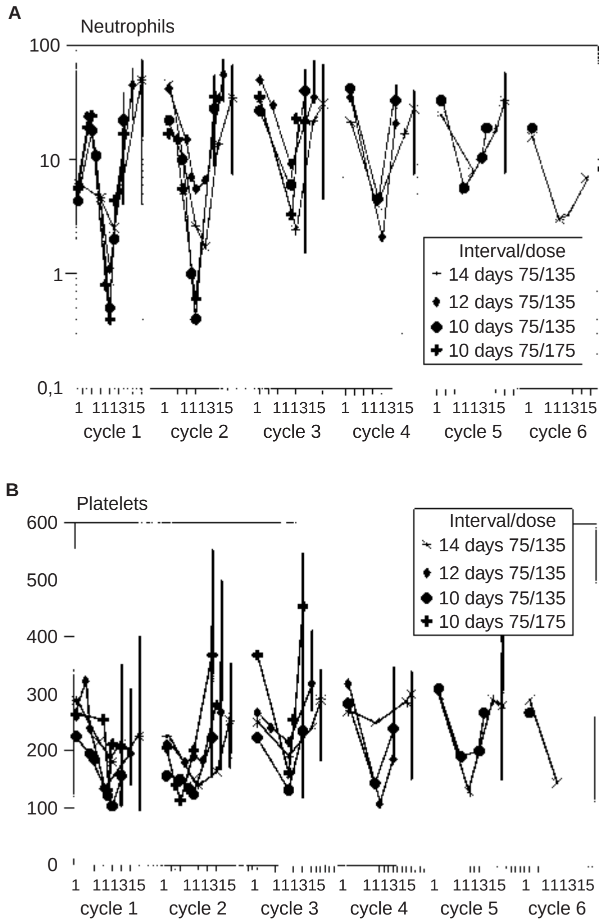

**Figure 1** Interval shortening of Epirubicin and Paclitaxel plus G-CSF. Median values per day and range of values at the day of next scheduled cycle, of consecutive chemotherapy cycles according to protocol: (**A**) neutrophils ($\times 10^9$ $l^{-1}$), (**B**) platelets ($\times 10^9$ $l^{-1}$)

ment may explain these transient DILCs. In the second patient (previously irradiated to the right chest wall) after three cycles in the 10-day interval at a dose of 75/175 mg $m^{-2}$ an asymptomatic decrease in LVEF (60→49%) was observed. The patient continued with three cycles of epirubicin/cyclophosphamide with complete normalization of the LVEF. From 20 patients cardiac evaluation was available with a cumulative epirubicin dose of ≥ 450 mg $m^{-2}$; median baseline LVEF 60% (range 50–72%) to 58% (range 30–70%). One patient in the 14-day interval (not previously irradiated) developed congestive heart failure with an LVEF of 30% after cycle 13 (cumulative epirubicin dose 975 mg $m^{-2}$). The single patient with a DILC in the 10-day interval at a dose of 75/135 mg $m^{-2}$ (previously irradiated to right chest wall and parasternal region) developed symptomatic ventricular extrasystoles after cycle 3, without a decrease in LVEF, and continued treatment for a total of six cycles. There was no real difference in median changes of LVEF from baseline and after chemotherapy between patients previously irradiated to the loco-regional breast/chest (27 patients, –4%, range –3– +16) and not irradiated patients (8 patients, –7%, range –24 – +9).

### Response rate

Although efficacy was not an aim of this study, formal UICC criteria were applied to the 29 patients with measurable disease. After three cycles, i.e. evaluation 20–42 days after the start of the study treatment an objective response (all partial) was already observed in 17 of 29 patients (59%, 95% CI, 41–77%), and there was one patient with progressive disease. Seventeen patients (stable disease 11 and partial response six) continued protocol treatment with an interval of ≤ 14 days for a median number of six cycles (range 4–9) whereby six additional patients reached a partial response and two partial responders reached a complete remission. The median interval between the start of therapy and the first observation of an objective response was 5 weeks (range 3–12 weeks). The best objective response (complete and partial) for the total study group was 79% (95% CI, 65–94%).

### DISCUSSION

The aim of our study was to determine the maximal dose intensity of epirubicin in combination with paclitaxel in a dose-dense schedule, supported by G-CSF. With a regimen consisting of epirubicin 75 mg $m^{-2}$ and paclitaxel 135 mg $m^{-2}$ an interval of 10 days was feasible, allowing a median DDI of 52 mg $m^{-2}$ per week for epirubicin and 94 mg $m^{-2}$ per week for paclitaxel, respectively. Within the 10-day interval it was feasible to increase the paclitaxel dose to 175 mg $m^{-2}$ enabling a median DDI of 122 mg $m^{-2}$ per week for paclitaxel in combination with 52 mg $m^{-2}$ per week of epirubicin. The treatment-schedule related toxicity was relatively mild, considering the high dose-intensity achieved.

The MTDs of the epirubicin and paclitaxel (3 h) combination in a 21-day schedule without 'prophylactic' haematopoietic growth factor as first-line treatment in metastatic breast cancer has been defined at 50/250 mg $m^{-2}$, 60/175 mg $m^{-2}$, 90/175 mg $m^{-2}$ and 90/200 mg $m^{-2}$, respectively. Dose-limiting toxicities were primarily haematological, namely severe neutropenia and febrile neutropenia (Catimel et al, 1996; Luck et al, 1997; Conte et al, 1997). The DDI in the latter study is 30 mg $m^{-2}$ per week for epirubicin and 67 mg $m^{-2}$ per week for paclitaxel, which is considerable lower than our results of 52 and 122 mg $m^{-2}$ per week, respectively.

**Table 4** Number of patients (percentage) encountering non-haematological toxicities (WHO-grading) for all cycles with epirubicin and paclitaxel with G-CSF support

| WHO grading | Nausea/ vomiting | Diarrhoea | Stomatitis | Neurotoxicity | Myalgia | Infection | Skin |
|---|---|---|---|---|---|---|---|
| 1 | 12 (32%) | 3 (8%) | 8 (22%) | 9 (24%) | 10 (27%) | 0 | 0 |
| 2 | 12 (32%) | 5 (14%) | 8 (22%) | 3 (8%) | 7 (19%) | 2 (5%) | 3 (8%) |
| 3 | 1 (3%) | 0 | 3 (8%) | 1 (3%) | 1 (3%) | 2 (5%) | 0 |
| 4 | 0 | 0 | 0 | 0 | 0 | 0 | 0 |

In a study of the epirubicin-paclitaxel combination in a 21-day interval without G-CSF the neutrophil-nadir occurs at day 12 and the median neutropenia grade 4 duration was 3 days (Conte et al, 1996). In our study, with G-CSF, the neutrophil-nadir was reached earlier, at day 8, was of very short duration, and was less pronounced in each consecutive cycle. G-CSF administered on all days, with the exception of the day of chemotherapy, hastened the neutrophil recovery after each chemotherapy course, but may also have had a pre-emptive effect on each consecutive cycle by expansion of the progenitor pool. This so-called pre-emptive G-CSF effect was, however, not observed earlier (Tjan-Heijnen et al, 1998; de Wit et al, 1996). Thrombocytopenia has not been dose-limiting. An important aspect of studies on escalated dose intensities is whether this can be maintained over repeated cycles. Conceivably with intervals as short as 10 days and retreatment at the moment of rapid recovery, the population of progenitor cells may be vulnerable to the repetitive cytotoxic insults and may become exhausted after a number of cycles. Although most data were collected over three cycles, we have not observed signs of exhaustion, even in the small number of patients treated up to six cycles. It may well be that treatment with G-CSF up to the day before the next chemotherapy has protected progenitor cells by putting them out of the cell cycle, as has been observed with GM-CSF (Kobrinsky et al, 1999; Vadhan-Raj et al, 1992). Further data on larger numbers of patients with even more cycles are needed to determine the absence of cumulative myelosuppression with certainty.

In the three epirubicin/paclitaxel studies the objective response rates after a least six cycles were 44%+, 68% (CR in 17%) and 84% (CR in 18%), respectively (Catimel et al, 1996; Luck et al, 1997; Conte et al, 1997). These data suggest a dose–response relationship for epirubicin. In our study, after three short interval cycles a response rate of 59% had already been observed.

An important argument to investigate epirubicin in combination with paclitaxel is to circumvent the increased cardiotoxicity of the doxorubicin/paclitaxel combination. After a median cumulative doxorubicin dose of 480 mg $m^{-2}$, 50% of the patients had reductions of the LVEF below the norm and 20% of the patients developed a congestive heart failure (Gianni et al, 1995*c*; Gehl et al, 1996). To prevent this excess cardiotoxicity the cumulative dose of doxorubicin in this combination needs to be limited to 360 mg $m^{-2}$, which makes this regimen not viable in patients pre-treated with doxorubicin containing ajuvant regimens (Hortobagyi et al, 1997). In the epirubicin/paclitaxel combination studies cardiac toxicity was observed in 13% (all anthracycline pre-treated), 4% and 6% of the patients, respectively (Catimel et al, 1996; Luck et al, 1997; Conte et al, 1997). These data and a recent article suggest less cardiotoxicity for the epirubicin/paclitaxel combination (Gennari et al, 1999).

Effective dose-dense combination chemotherapy with short intercyclic intervals affords less opportunity for the emergence and regrowth of drug-resistant cell clones, a premise upon which this concept is based (Gilewski and Norton, 1996). Our schedule may have advantages with respect to both sustained exposure and dose-dense drug delivery.

In conclusion, with the addition of G-CSF, shortening of intervals of chemotherapy seems to be an effective method of dose intensification, allowing a dose-intensity of 52 mg $m^{-2}$ per week of epirubicin in combination with 122 mg $m^{-2}$ per week of paclitaxel. The efficacy and clinical relevance of this approach is now being investigated in a phase II study.

### ACKNOWLEDGEMENTS

The study was supported in part by Bristol-Myers Squibb, Amgen-Roche and Pharmacia & Upjohn (all The Netherlands). We thank JW Wagstaff MD PhD for comments on the paper and A Epping, M Bongers, M Jansen, H Leurs and A Kroen for their assistance in data collection.

### REFERENCES

Brambilla C, Rossi A, Bonfante V, Ferrari L, Villani F, Crippa F and Bonadonna G (1986) Phase II study of doxorubicin versus epirubicin in advanced breast cancer. *Cancer Treatment Report* **70**: 261–266

Catimel G, Spielmann M, Dieras V, Kayitalire L, Pouillart P, Guastalla JP, Soler-Michel P, Graffand N, Garet F, Dumortier A, Pellae-Cosset B and Chazard M (1996) Phase I study of paclitaxel and epirubicin in patients with metastatic breast cancer: a preliminary report on safety. *Semin Oncol* **23** (Suppl 1): 24–27

Conte PF, Michelotti A, Baldini E, Salvadori B, Gennari A, Da Prato M, Tibaldi C, Salzano E and Gentile A (1996) A dose-finding study of epirubicin in combination with paclitaxel in the treatment of advanced breast cancer. *Semin Oncol* **21** (Suppl 11): 28–31

Conte PF, Baldini E, Gennari A, Michelotti A, Salvadori B, Tibaldi C, Danesi R, Innocenti F, Gentile A, Dell'Anna R, Biadi O, Mariani M and Del Tacca M (1997) Dose-finding study and pharmacokinetics of epirubicin and paclitaxel over 3 hours: a regimen with high activity and low cardiotoxicity in advanced breast cancer. *J Clin Oncol* **15**: 2510–2517

de Wit R, Verweij J, Bontenbal M, Kruit WH, Seynaeve C, Schmitz PI and Stoter G (1996) Adverse effect on bone marrow protection of prechemotherapy granulocyte colony-stimulating factor support [see comments]. *J Natl Cancer Inst* **88**: 1393–1398

Eisenhauer EA, ten Bokkel Huinink WW, Swenerton KD, Gianni L, Myles J, van der Burg ME, Kerr I, Vermorken JB, Buser K and Colombo N (1994) European-Canadian randomized trial of paclitaxel in relapsed ovarian cancer: high-dose versus low-dose and long versus short infusion. *J Clin Oncol* **12**: 2654–2666

Fisherman JS, McCabe M, Noone M, Ognibene FP, Goldspiel B, Venzon DJ, Cowan KH and O'Shaughnessy JA (1993) Phase I study of Taxol, doxorubicin, plus granulocyte-colony stimulating factor in patients with metastatic breast cancer. *J Natl Cancer Inst Monograph*: 189–194

Gehl J, Boesgaard M, Paaske T, Vittrup Jensen B and Dombernowsky P (1996) Combined doxorubicin and paclitaxel in advanced breast cancer: effective and cardiotoxic. *Ann Oncol* **7**: 687–693

Gennari A, Salvadori B, Donati S, Bengala C, Orlandini C, Danesi R, Del Tacca M, Bruzzi P and Conte PF (1999) Cardiotoxicity of epirubicin/paclitaxel-containing regimens: role of cardiac risk factors. *J Clin Oncol* **17**: 3596–3602

Gianni L, Munzone E, Capri G, Villani F, Spreafico C, Tarenzi E, Fulfaro F, Caraceni A, Martini C and Laffranchi A (1995*a*) Paclitaxel in metastatic breast cancer: a trial of two doses by a 3-hour infusion in patients with disease recurrence after prior therapy with anthracyclines. *J Natl Cancer Inst* **87**: 1169–1175

Gianni L (1995*b*) Theoretical and practical aspects of paclitaxel scheduling. *Ann Oncol* **6**: 861–863

Gianni L, Munzone E, Capri G, Fulfaro F, Tarenzi E, Villani F, Spreafico C, Laffranchi A, Caraceni A and Martini C (1995*c*) Paclitaxel by 3-hour infusion in combination with bolus doxorubicin in women with untreated metastatic breast cancer: high antitumor efficacy and cardiac effects in a dose-finding and sequence-finding study. *J Clin Oncol* **13**: 2688–2699

Gilewski TA and Norton L (1996) Cytokinetics and breast cancer chemotherapy. In: *Diseases of the Breast*, Harris JR, Lippman ME, Morrow M and Hellman S (eds) pp 751–768. Lippincott-Raven: Philadelphia

Henderson IC, Hayes DF and Gelman R (1988) Dose-response in the treatment of breast cancer: a critical review. *J Clin Oncol* **6**: 1501–1515

Hortobagyi GN, Willey J, Rahman Z, Holmes FA, Theriault RL and Buzdar AU (1997) Prospective assessment of cardiac toxicity during a randomized phase II trial of doxorubicin and paclitaxel in metastatic breast cancer. *Semin Oncol* **24** (Suppl 17): 65–68

Jain KK, Casper ES, Geller NL, Hakes TB, Kaufman RJ, Currie V, Schwartz W, Cassidy C, Petroni GR and Young CW (1985) A prospective randomized comparison of epirubicin and doxorubicin in patients with advanced breast cancer. *J Clin Oncol* **3**: 818–826

*© 2000 Cancer Research Campaign*

Kobrinsky NL, Sjolander DE, Cheang MS, Levitt R and Steen PD (1999) Granulocyte-macrophage colony-stimulating factor treatment before doxorubicin and cyclophosphamide chemotherapy priming in women with early-stage breast cancer. *J Clin Oncol* **17**: 3426–3430

Lalisang RI, Wils JA, Nortier HW, Burghouts JT, Hupperets PS, Erdkamp FL, Schouten HC and Blijham GH (1997) Comparative study of dose escalation versus interval reduction to obtain dose-intensification of epirubicin and cyclophosphamide with granulocyte colony-stimulating factor in advanced breast cancer. *J Clin Oncol* **15**: 1367–1376

Luck HJ, Thomssen C, du Bois A, Untch M, Lisboa B, Kohler G and Diergarten K (1997) Phase II study of paclitaxel and epirubicin as first-line therapy in patients with metastatic breast cancer. *Semin Oncol* **24** (Suppl 17): 35–39

Miller AB, Hoogstraten B, Staquet M and Winkler A (1981) Reporting results of cancer treatment. *Cancer* **47**: 207–214

Nabholtz JM, Gelmon K, Bontenbal M, Spielmann M, Catimel G, Conte P, Klaassen U, Namer M, Bonneterre J, Fumoleau P and Winograd B (1996) Multicenter, randomized comparative study of two doses of paclitaxel in patients with metastatic breast cancer (see comments). *J Clin Oncol* **14**: 1858–1867

Sledge Jr GW, Robert N, Sparano JA, Cobeligh M, Goldstein LJ, Neuberg D, Rowinsky E, Baughman C and McCaskill-Stevens W (1994) Paclitaxel (Taxol)/doxorubicin combinations in advanced breast cancer: the Eastern Cooperative Oncology Group experience. *Semin Oncol* **21**: 15–18

Tjan-Heijnen VC, Biesma B, Festen J, Splinter TA, Cox A, Wagener DJ and Postmus PE (1998) Enhanced myelotoxicity due to granulocyte colony-stimulating factor administration until 48 hours before the next chemotherapy course in patients with small-cell lung carcinoma (see comments). *J Clin Oncol* **16**: 2708–2714

Vadhan-Raj S, Broxmeyer HE, Hittelman WN, Papadopoulos NE, Chawla SP, Fenoglio C, Cooper S, Buescher ES, Frenck RWJ and Holian A (1992) Abrogating chemotherapy-induced myelosuppression by recombinant granulocyte-macrophage colony-stimulating factor in patients with sarcoma: protection at the progenitor cell level. *J Clin Oncol* **10**: 1266–1277

*© 2000 Cancer Research Campaign* 
